# Untargeted Metabolomics Approach for the Differentiation between *Panax vietnamensis* var. *vietnamensis* and *Panax vietnamensis* var. *fuscidiscus*

**DOI:** 10.3390/metabo13060763

**Published:** 2023-06-19

**Authors:** Huy Truong Nguyen, Long Ke Phan, Kim-Long Vu Huynh, Thuc-Huy Duong, Huong Thuy Le, Nguyen Trang Hai-Yen, Nguyen Thi Hai Yen, Nguyen Phuoc Long, Minh Duc Nguyen

**Affiliations:** 1 Faculty of Pharmacy, Ton Duc Thang University, Ho Chi Minh City 700000, Vietnam; nguyentruonghuy@tdtu.edu.vn (H.T.N.); vuhuynhkimlong@tdtu.edu.vn (K.-L.V.H.); lethuyhuong@tdtu.edu.vn (H.T.L.); h1800361@student.tdtu.edu.vn (N.T.H.-Y.); 2 Vietnam National Museum of Nature, Vietnam Academy of Science and Technology, Hanoi 122300, Vietnam; pklong@vnmn.vast.vn; 3 Department of Chemistry, University of Education, Ho Chi Minh City 72700, Vietnam; huydt@hcmue.edu.vn; 4 Department of Pharmacology and Pharmaco Genomics Research Center, Inje University College of Medicine, Busan 47392, Republic of Korea; 2021b171@live.inje.ac.kr

**Keywords:** Vietnamese ginseng, *Panax vietnamensis* var. *vietnamensis*, *Panax vietnamensis* var. *fuscidiscus*, untargeted metabolomics, authentication

## Abstract

*Panax vietnamensis* var. *vietnamensis* (PVV) and *Panax vietnamensis* var. *fuscidiscus* (PVF) both belong to *Panax vietnamensis* species and are chemically and morphologically similar, making it hard to distinguish for the consumer. Herein, 42 PVF and 12 PVV samples were collected in Quang Nam and Lai Chau Province, respectively, and subsequently characterized by ITSr-DNA sequence data to verify their origins. Next, untargeted metabolomics combined with multivariate statistical analysis was developed to differentiate PVV and PVF. The metabolic profiles of PVV and PVF were found to be distinct and classified well using Partial Least-Squares Discriminant Analysis (PLS-DA) in the training set. Among them, seven ginsenosides were of high abundance in PVV, while six were of high abundance in PVF. Next, the test set was used to validate 13 putative differential markers found in the training set, illustrating a complete match with the expression patterns of these ginsenosides in the training set. Finally, PLS-DA and linear Support Vector Machine models both indicated distinct ginsenoside profiles of PVV and PVF without misclassification in the test set. Conclusively, the developed untargeted metabolomics approach might serve as a powerful tool for the authentication of PVV and PVF at the metabolome level.

## 1. Introduction

**Figure 1:**
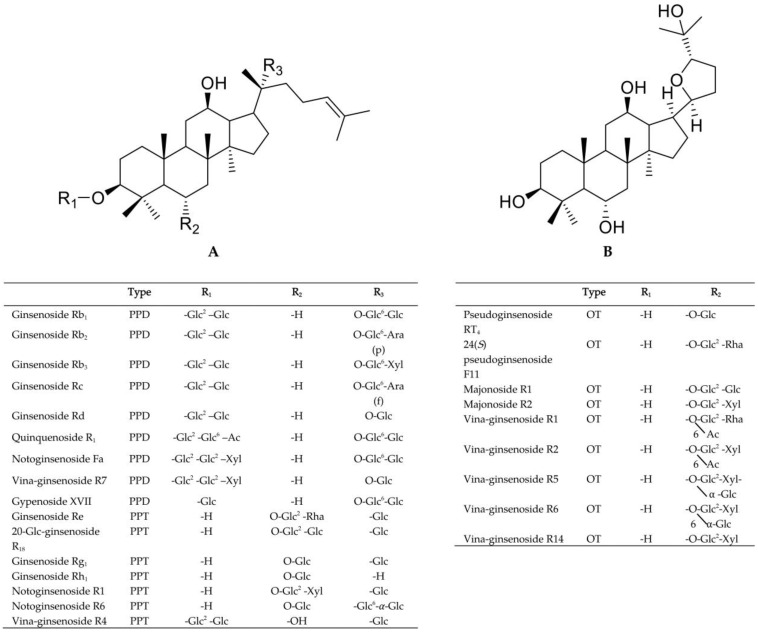
Structure of some saponins isolated from *P. vietnamensis* var. *vietnamensis*.

*Panax vietnamensis* var. *vietnamensis* (PVV), the so-called Vietnamese ginseng, is one of the recent members of the *Panax* species of the Araliaceae family and has been traditionally utilized for its medicinal purposes by the Se Dang ethnic people in Quang Nam Province, Vietnam [1]. It is considered an endemic species and a “national treasure” by the Vietnamese government. Since its initial discovery in 1973, PVV has garnered increasing attention from the scientific community. Numerous studies indicate that PVV’s chemical profiles share similarities in protopanaxadiol-type saponins (PPD-type saponins) and protopanaxatriol-type saponins (PPT-type saponins) with other *Panax* species such as *Panax ginseng*, *Panax notoginseng*, etc. However, what makes PVV unique is the ocotillol-type saponins (OT-type saponins), which account for nearly more than fifty percent of PVV’s total saponin content (Figure 1) [2,3,4]. In particular, majonoside-R2, a major compound with a content of over 5%, indicated notable biological effects, such as anti-tumor activity in a two-stage carcinogenesis test in mouse models, protective activity against free radical-induced tissue injury in vitro, suppressive effects on psychological stress in mice models, etc. In terms of therapeutic value, PVV possesses a number of intriguing biological activities, including anti-melanogenic, anti-oxidative, anti-stress, anti-cancer, hepato-cytoprotective, and nephroprotective effects against cisplatin toxicity [5,6,7,8,9,10,11,12].

Interestingly, in China, a new variety of *Panax vietnamensis*, namely *Panax vietnamensis* var. *fuscidiscus* (PVF), was discovered by Zhu et al. in 1993 [13]. Additionally, it was also preliminarily reported that PVF possessed greatly similar morphological characteristics, as well as chemical constituents to that of PVV, with more than fifty percent of its total saponins being ocotillol-type saponins, specifically M-R2 and vinaginsenoside R2 (V-R2) [14]. It was not until 2013 that the first occurrence of PVF was reported in Lai Chau Province, Vietnam, by Phan et al., in terms of morphological characteristics, ecology, and natural conditions [15]. Therefore, currently in the market, there are two available sources of Vietnamese ginseng: PVV originating from Ngoc Linh mountainous area in Quang Nam and Kon Tum Province and PVF originating from Lai Chau Province and mostly from China. However, in the practice of grading and pricing Vietnamese ginseng, the origin plays a crucial role. In fact, PVV is much better regarded by the market, resulting in much higher economic value than PVF. Thus, the high demand for PVV, along with the difficulties in the morphological differentiation between PVV and PVF owing to their remarkable similarity, lead to intentional and/or unintended adulterations. Such practices might be due to the addition and/or substitution of PVF with PVV or to unintentional misidentification of PVV. Therefore, an effective authentication tool between PVV and PVF is a pressing need of the market.

Up to now, for the purpose of differentiation of *Panax* species, DNA-based approaches take advantage of the higher thermal stability of DNA molecules and their independence from physiological and external conditions and are regarded as powerful tools for botanical identification of plant species in a wide variety of foods, including herbal supplements. Among DNA-based techniques, DNA barcoding has demonstrated significant utility in the botanical authentication of a wide variety of medicinal plants. Indeed, many DNA-based approaches have successfully authenticated PVF from PVV [16,17,18]. Nevertheless, methods that can differentiate between PVV and PVF based on their ginsenoside profiling are still very much needed, because when it comes to the quality of ginseng, the ginsenoside profiles are a vital factor. Therefore, clarifying the ginsenoside compositions of each used plant is a crucial aspect of authenticating herbs that are readily confounded. Attractive current topics in the field of analytical chemistry include the development of effective and practical methods for the comprehensive deconvolution of plant metabolites from closely related species. Ultra-high performance liquid chromatography-quadrupole time-of-flight mass spectrometry (UPLC-QTOF-MS) profiling coupled with chemometrics can make untargeted metabolomics analysis suitable for systematically identifying potential chemical markers for authentication.

The aim of this work was to develop an untargeted metabolomics approach, by UPLC-QTOF-MS, to systematically investigate the metabolome difference between PVV and PVF. Forty-two whole roots of PVF and 17 whole roots of PVV were collected at Lai Chau Province and Kon Tum Province, respectively. Then, three representative samples of each group were characterized using ITSr-DNA sequence data to verify their origins. As for the UPLC-QTOF-MS analysis, the conditions for chromatographic separation and MS detection were optimized to enable the resolution and sensitive monitoring of the multicomponent. Forty-two samples (30 PVF and 12 PVV, training set) were used for the discovery of putative ginsenoside biomarkers. The putative biomarkers found in the training set were validated in a test set comprised of 17 samples (12 PVF and 5 PVV). Chemometrics and machine learning modeling were applied to discover potential markers, further identified by multiple approaches, such as comparison with the reference compounds, and searching against an in-house ginsenoside library. The results obtained could clearly be well distinguished between PVV and PVF, employing 13 markers from the utilized machine learning models. Among them, seven were of high abundance in the PVV, including majonoside R2 (M-R2), vinaginsenoside R13 (V-R13), ginsenoside Rd (G-Rd), ginsenoside Rb1 (G-Rb1), notoginsenoside Fa (N-Fa), pseudoginsenoside Rs1 (PG-Rs1) and quinquenoside R1 (Q-R1). On the contrary, six were of high abundance in the PVF, including majonoside R1 (M-R1), vinaginsenoside R2 (V-R2), ginsenoside Rb2 (G-Rb2), notoginsenoside Fc (N-Fc), notoginsenoside R2 (N-R2), and notoginsenoside R4 (N-R4). To the best of our knowledge, this is the first report highlighting the differences between the ginsenoside profiles of PVV and PVF and their corresponding differential markers, thus establishing valuable information for the authentication of these two valuable ginsengs.

## 2. Materials and Methods

### 2.1. Reagents and Material

PVV roots (*n* = 17) (Figure S1) were donated by VINGIN JSC, a Vietnamese ginseng farm at Ngoc Linh Mountain, Kon Tum Province, while PVF roots (*n* = 42) (Figure S2) were harvested in Muong Te commune, Lai Chau Province and supplied by ThuKa local cooperative in Phong Tho commune. The three representative samples of PVV and PVF groups were characterized as PVV and PVF by Prof. Phan Ke Long using ITS-rDNA gene sequencing. All the fresh roots were dried at 60 °C until dryness before reducing to fine powder with particle size under 425 µm. LC-MS grade acetonitrile and methanol were purchased from J. T. Baker (Radnor, PA, USA). MS grade formic acid and water were provided by Merck (Louis, MO, USA). Specimens were deposited at the authors’ lab in Ton Duc Thang University (Ho Chi Minh City, Vietnam).

### 2.2. Sample Preparation

An aliquot of 100 mg sample powder was placed into a 15 mL test tube. Then, an exact amount of 10 mL of 70% methanol was added to the sample. The test tube was capped tightly, and the mixture was extracted by sonication for 40 min. The extract was centrifuged at 14,000 rpm for 10 min, and the supernatant was used for the test sample. An equal amount of 0.5 mL from each sample was mixed to create one pooled quality control (QC) sample. The samples were filtered through a 0.22 µm filter prior to LC-MS analysis.

### 2.3. UPLC-QTOF-MS Analysis

The data acquisition was carried out in an Agilent Infinity 1290 system coupled with a 6456 QTOF detector. The separation was archived using Agilent EclipsePlus C18 RRHD (2.1 × 50 mm, 1.8 µm). The mobile phase composed of acetonitrile (A) and water (B) containing 0.1% of formic acid in both channels with the gradient elution was as follows: 0–23 min: 17–18% A; 23–33 min: 18–27% A; 33–47 min: 27–28% A; 47–50 min: 28–40% A; 50–55 min: 40–95% A. The column was reconditioned with starting mobile phase for 2 min before analyzing a new sample. The flow rate was kept at 0.3 mL/min, and the injection volume was 2.00 µL. The TOF-MS was carried out in negative mode with the range of scan *m/z* 100–1700 Da. The gas temperature was set at 300 °C with gas flow of 5 L/min. The sample was ionized at the fragmentor of 350 V, and the capillary voltage was 3500 V.

A randomized sequence was created for the data acquisition. The acquisition started and ended with the injection of several blank samples that would later also be used for data processing. Six QC samples were first injected for LC and MS equilibration. Then, we injected 2 QC samples for every 8 samples (before and after), except for the final pairs, which contained only 4 samples in between.

### 2.4. UPLC-QTOF-MS Data Processing and Normalization

The *.d files were imported directly to MS-DIAL for data processing [19]. For data collection, an MS1 tolerance of 0.01 Da was utilized. Peak detection was conducted using linear weighted moving average as the smoothing method (smoothing level of 3 scans and minimum peak width, 3 scans) with the minimum peak height of 1000 amplitude and mass slice width of 0.1 Da. Since alignment is a crucial step that critically influences the quality of the subsequent analyses, the following stringent parameters were set: retention time tolerance (across samples), 0.05 min; MS1 tolerance, 0.025 Da; retention time factor, 0.5; MS1 factor, 0.5; peak count filter, 30%; remove features based on blank information activated, sample average/blank average, 5 folds.

Automated ginsenoside annotation was conducted using our in-house *m/z* and RT database (accurate mass tolerance of 0.01 da, retention time tolerance of 0.3 min, and identification score cut-off of 70%). Manual inspection was also conducted by at least two experienced researchers to guarantee the accuracy of the ginsenoside annotation. The annotation results can be found in Table S1.

The final processed data were partitioned into a training set (70%) and a test set (30%). The data partition was conducted using the caret R package. The training and test sets were separately subjected to further treatment and normalization. In brief, we removed all features with >25% missing values. The remaining features with missing values were imputed using kNN (feature-wise) method. It is of importance to note that missing values commonly happen in LC-MS-based untargeted metabolomics data. However, most of the statistical and machine learning methods require a complete data set [20]. Hence, removing features with a substantial portion of the missing value (i.e., >25%) and imputing the remaining features are crucial steps. Next, features with a relative standard deviation (of the QC samples) >20% were discarded. The data were then normalized by median, log (base 10) transformed, and Pareto scaled before the subsequent analyses.

### 2.5. Multivariate and Machine Learning Analyses

Unsupervised techniques, Principal Component Analysis (PCA) and heatmap visualization (with sample-wise and/or feature-based clustering) were applied to the whole data set to explore the characteristics and tendency of clustering of the samples belonging to PVV and PVF groups. The *t*-test was applied to discover potential biomarker features. In addition, Partial Least-Squares Discriminant Analysis (PLS-DA) was also applied for classification. The cross-validation of PLS-DA was conducted. The VIP score of the first component of the optimal PLS-DA model was also extracted. The false-discovery rate (FDR) of 0.01 was used as the statistically significant threshold of the *t*-test. A Q^2^ of 0.4 was considered a predictive PLS-DA model, and a VIP score of 1 or higher indicated a significant contribution a variable makes to the model. Since we focused on the ginsenosides as potential biomarkers for PVV and PVF, the VIP score was considered additional evidence for the relative importance of identified ginsenosides. The ROC curve-based model evaluation was conducted on the test set using biomarker candidates found in the training set. The machine learning algorithms used were PLS-DA and linear support vector machines (SVM). PLS-DA and linear SVM are the two commonly used algorithms in metabolomics. We checked the performance of PLS-DA and SVM regarding the classification between PVV and PVF [21]. All analyses were implemented in MetaboAnalyst 5.0 [22]. The study workflow was visualized in Figure S3.

### 2.6. Assessment of Sample Using ITS-rDNA Sequence Method

DNA extraction and purification: Total DNA was extracted and purified using the Plant DNA Isolation Kit (Norgenbiotek, Thorold, ON, Canada).

PCR amplification: The ITS-rDNA regions were amplified using PCR technique according to Phan et al. (2014) with primer pairs PaITSF 5′-CAC TGA ACC TTA TCA TTT AGA G-3′ and PaITSR 5′-CTT ATT GAT ATG CTT AAA CTC AG-3′. The composition of each PCR reaction has a volume of 25 µL with the following components: 7 µL deionized H_2_O (18 MΩ); 12.5 µL PCR Master mix kit (2X); 1.25 µL forward primer (10 pmol/µL); 1.25 µL reverse primer (10 pmol/µL); 3 µL DNA (10–20 ng). The reaction was performed on a model 9700 PCR machine (GeneAmp PCR System 9700, Foster City, CA, USA). The thermal cycle of the PCR reaction was: 94 °C for 3 min, followed by 35 consecutive cycles with steps: 94 °C for 45 s, 49 °C for 45 s, and 72 °C for 45 s, respectively; end the gene multiplication reaction at 72 °C for 10 min, keeping the product at 4 °C.

Sequencing and data analysis: The nucleotide sequencing was performed at the Institute of Biotechnology—Vietnam Academy of Science and Technology. The DNA sequence after sequencing was corrected and regions of noise were removed using ChromasPro2.1.6 software. Nucleotide sequences of ginseng samples were compared with those available on GenBank (using the BLAST tool in NCB—http:www.ncbi.nlm.nih.gov/BLAST (accessed on 11 May 2023)). The phylogenetic relationship between specimens with other species in the Panax genus was created using Mega 11 using maximum likelihood method (ML) [23].

Genbank accession number: The ITS-rDNA sequence length was compared with the submitted sequence of *P. vietnamensis* var. *vietnamensis* (Genbank accession number KJ418193); *P. vietnamensis* var. *fuscidiscus* Muong Te population (Genbank accession number KJ41891); *P. vietnamensis* var. *fuscidiscus* Sin Ho and Tam Duong population (Genbank accession number KJ418187).

## 3. Results and Discussion

### 3.1. Authenticating the Origins of Vietnamse Ginseng by ITSr-DNA Sequence Data

**Figure 2:**
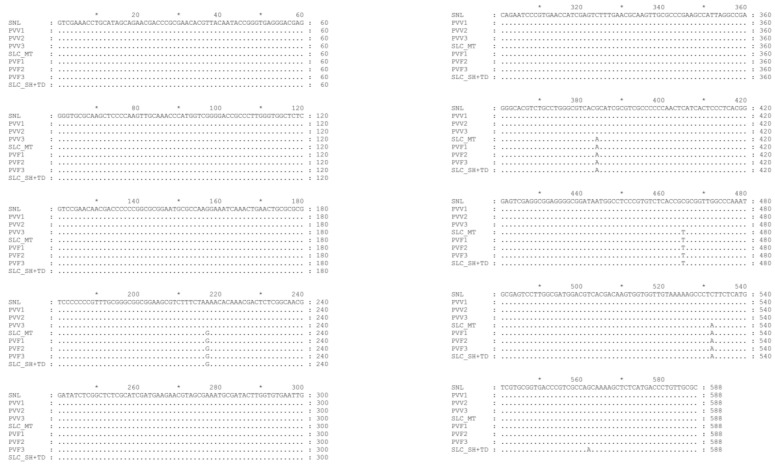
The comparison of all the representative samples from each group. SNL: *P. vietnamensis* var. *vietnamensis* (KJ418193). SCL_MT: *P. vietnamensis* var. *fuscidiscus* Muong Te population (KJ418191). SLC_SH+TD: *P. vietnamensis* var. *fuscidiscus* Sin Ho and Tam Duong population (KJ418187). The star symbol indicated the even tens of nucleotides.

The ITS-rDNA sequence length of all representative samples from each group was 588 bp and had a close relationship with *P. vietnamensis* var. *vietnamensis* and *P. vietnamensis* var. *fuscidiscus* in which the ITS-rDNA of PVV1, PVV2, and PVV3 samples were identical with *P. vietnamensis* var. *vietnamensis* (Genbank accession number KJ418193) and PVF1, PVF2, and PVF3 samples were identical with *P. vietnamensis* var. *fuscidiscus* Muong Te population (Genbank accession number KJ41891) (Figure 2). Additionally, in the phylogenetic tree (Figure S4), the specimens PVV1-PVV3 were clustered with *P. vietnamensis* var. *vietnamensis* (KJ418193) and PVF1-PVF3 were clustered with *P. vietnamensis* var. *fuscidiscus* which originated from Muong Te district (KJ418191).

Based on this analysis, we confirmed that the samples PVV1, PVV2, and PVV3 were *P. vietnamensis* var. *vietnamensis* and the samples PVF1, PVF2, and PVF3 were *P. vietnamensis* var. *fuscidiscus* Muong Te population. Consequently, this experiment proved that all the samples were true to their origins.

### 3.2. Optimization of UPLC-QTOF-MS Approach for the Ginsenosides Profiling

The UPLC-TOF-MS condition was optimized in terms of MS detection parameters and gradient elution program. To improve the method’s sensitivity, the result showed that the increase of fragmentor also increased the peak height, and the value of 350 V led to the highest signal intensity.

The gradient elution program was also optimized, including the gradient elution program and the addition of formic acid as solvent modifier. The decrease in the initial acetonitrile concentration from 19% to 17% could result in the excellent separation of the early elute peaks such as M-R1 and N-R1; G-Rg1, M-R2, and vinaginsenoside R11 (V-R11). Notably, the separation of M-R2 and V-R11 was essential due to the similar mass of these two compounds (*m*/*z* 785.47). The prolonged increase of acetonitrile content from 27–28% for 15 min (33–47 min) could separate the protopanaxadiol peaks’ complex peak profile. Although the MS detector could differentiate the peaks by mass value, the good separation of the peaks could visually express the difference in chemical composition in these two varieties.

Regarding the solvent modifier, mobile phase without formic acid could give a noisy baseline. Adding this mobile phase modifier agency could significantly reduce the background noise. The addition of formic acid increase the ionization of the saponins leading to a higher peak intensity and thereby improving the sensitivity of the analysis.

**Figure 3:**
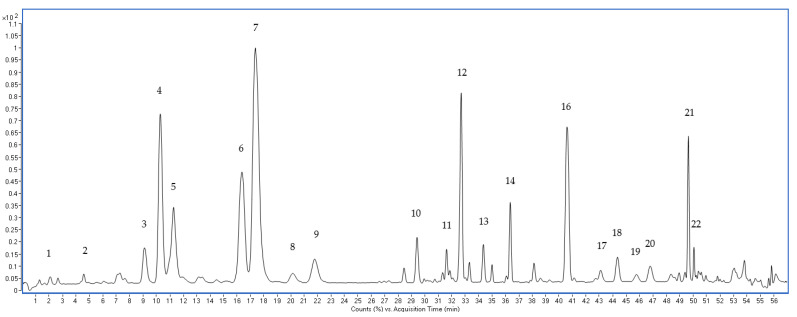
Representative chromatogram of QC samples analyzed by LC-TOF-MS. 1: Vinaginsenoside R13; 2: Vinaginsenoside R14; 3: Vinaginsenoside R4; 4: Majonoside R1; 5: Notoginsenoside R1; 6: Ginsenoside Rg1; 7: Majonoside R2; 8: Pseudoginsenoside Rt4; 9: Vinaginsenoside R11; 10: Hemsloside Ma3; 11: Pseudoginsenoside Rc1; 12: Vinaginsenoside R2; 13: Notoginsenoside R2; 14: Notoginsenoside R4; 15: Notoginsenoside Fa; 16: Ginsenoside Rb1; 17: Quinquenoside R1; 18: Notoginsenoside Fc; 19: Quinquenoside R1 isomer; 20: Ginsenoside Rb2; 21: Ginsenoside Rd; 22: Pseudoginsenoside Rs1.

The representative UPLC-TOF chromatogram of QC sample at optimized conditions is shown in Figure 3.

### 3.3. Multivariate Analysis of the Metabolome Differences between PVV and PVF

**Figure 4:**
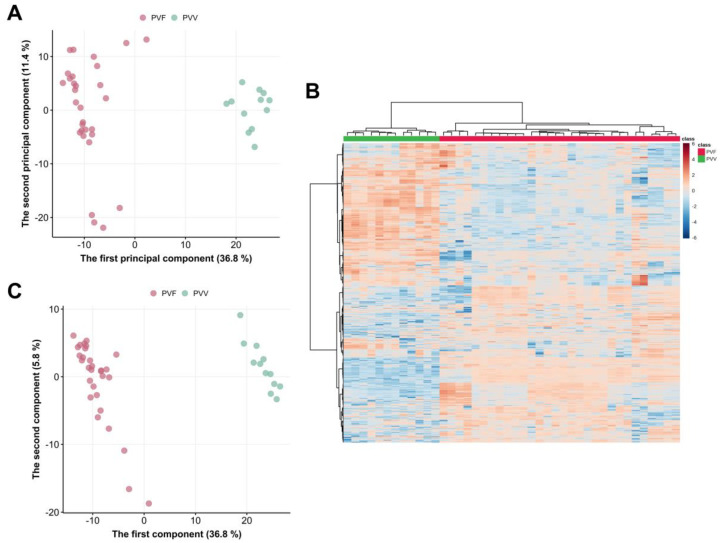
Multivariate analysis for the tendency of difference of the metabolome between PVV and PVF. (**A**) Principal Component Analysis, (**B**) Partial Least-Squares Discriminant Analysis (PLS-DA), (**C**) Heatmap with sample-wise and feature-based clustering using the whole list of reliably detected features.

We next conducted the exploratory data analysis on the training set using heatmap (with feature-based clustering) and PCA. As shown in Figure 4A, PC1 and PC2 made up a total explained variance of 48.2%, while PC1 alone contained 36.8%. The PCA scores plot indicated a clear separation of samples belonging to PVV and PVF. These suggested that the metabolic features of the two groups were different. The heatmap further supported this observation that the samples formed two clusters without mismatching (Figure 4B).

**Figure 5:**
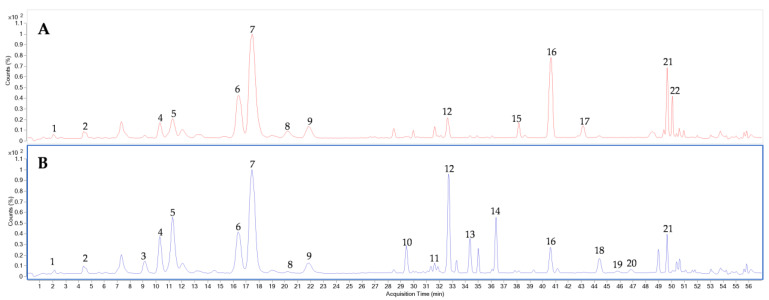
Representative chromatogram of PVV (**A**) and PVF (**B**). 1: Vinaginsenoside R13; 2: Vinaginsenoside R14; 3: Vinaginsenoside R4; 4: Majonoside R1; 5: Notoginsenoside R1; 6: Ginsenoside Rg1; 7: Majonoside R2; 8: Pseudoginsenoside Rt4; 9: Vinaginsenoside R11; 10: Hemsloside Ma3; 11: Pseudoginsenoside Rc1; 12: Vinaginsenoside R2; 13: Notoginsenoside R2; 14: Notoginsenoside R4; 15: Notoginsenoside Fa; 16: Ginsenoside Rb1; 17: Quinquenoside R1; 18: Notoginsenoside Fc; 19: Quinquenoside R1 isomer; 20: Ginsenoside Rb2; 21: Ginsenoside Rd; 22: Pseudoginsenoside Rs1.

PLS-DA clearly differentiated samples from PVV and PVF (accuracy, 100%; R^2^, 1.00; Q^2^, 0.98) (Figure 4C). Additionally, there were 875 significant features with the FDR cut-off of 0.01 in the *t*-test. Out of the significant features, 13 were annotated as ginsenosides. Among significantly different ginsenosides, seven were of high abundance in the PVV, although their VIP scores of some ginsenosides were <1: pseudoginsenoside Rs1 (PG-Rs1, VIP = 1.49), N-Fa (VIP = 1.37), Q-R1 (VIP = 1.30), G-Rd (VIP = 0.87), G-Rb1 (VIP = 0.71), V-R13 (VIP = 0.62), and M-R2 (VIP = 0.41). On the contrary, six were of high abundance in the PVF. These were N-Fc (VIP = 2.07), N-R2 (VIP = 1.65), G-Rb2 (VIP = 1.62), V-R2 (VIP = 1.58), N-R4 (VIP = 2.09), and M-R1 (VIP = 0.84) (Table 1). Among these markers, G-Rd, G-Rb1, G-Rb2, Q-R1, N-R4, N-Fa, and N-Fc are PPD-type saponins; N-R1 and PG-Rs1 are PPT-type saponins; and M-R2, M-R1, V-R2, and V-R13 are OT-type saponins. It could be noted that PVV differs from PVF in various ginsenosides belonging to all typical saponin types. Zhu et al. has observed in their preliminary report on PVF that OT-type saponins might be the differential markers, and a complicated pattern of unidentified peaks in medium polar region to the non-polar region of the chromatogram of C18 column could also well distinguish PVV from PVF [14]. Here, in our study, we confirmed four similar markers by reference standards, for example, OT-type saponins such as V-R2 and M-R2, and PPT-type saponins such as G-Rb1 and G-Rd. Therefore, an absolute quantitative assay of these markers should be expected to give a better view of the differences in their contents. It is also worth mentioning that other tentative identified markers, including N-Fa, N-Fc, N-R2, N-R4, PG-Rs1, and Q-R1 were eluted in the medium to non-polar region of the chromatogram and reflected well the differences between PVV and PVF (Figure 5). Interestingly, these tentative markers have rarely been utilized for the quality control of *Panax* species in general. Thus, it is suggested that further isolation, purification, and confirmation of these tentative markers should be performed in the future with the help of phytochemical isolation and Nuclear Magnetic Resonance analyses, resulting in the establishing of reference standards for the potential quality control of PVV and PVF. Consequently, the insignificant contributions of some ginsenosides to the PLS-DA model could be associated with the fact that PVV and PVF chemical profiles were different due to many other metabolites rather than ginsenosides alone (Figure 4B), suggesting new targets for follow-up studies.

Additionally, as can be observed from the phylogenetic tree (Figure S3), the closest taxonomic relatives of the *P. vietnamensis* species are *P. japonicus* var. *bipinnatifidus* and *P. pseudoginseng* var. *bipinnatifidus*, which so far have few reports on their saponin composition. However, according to those few reports, among those identified markers, N-R1, N-R2, G-Rb1, and G-Rd have been reported in *P. japonicus* var. *bipinnatifidus* [14], while G-Rb2, G-Rb1, and G-Rd have been reported in *P. pseudoginseng* var. *bipinnatifidus* [24]. Other identified markers, particularly OT-type saponins including M-R2, V-R2, V-R13, and M-R1 thus far have been reported only in *P. vietnamensis* species. Therefore, it is suggested that OT-type saponins are not only useful for differentiating *P. vietnamensis* as the species level from its close phylogenetic taxonomic relatives but also crucial for distinguishing *Panax vietnamensis* at its varieties level.

Among the markers, OT-type saponins such as M-R2 and V-R2 have been known mainly for their anti-inflammatory effects [25], while other PPD-type saponins such as G-Rd, G-Rb1, G-Rb2, N-Fa, N-Fc and PPT-type saponins such as N-R1 have been reported with various pharmacological activities, including anti-amnestic and anti-aging [26], neuroprotective [27], reduction of renal tubular injury and mitochondrial dysfunction [28], anti-hyperlipidemic activities [29], anti-coagulation [30], etc. Usually, the differences in the metabolite composition of the herbal medicine are directly related to the differences in their clinical efficacy. Hence, it is expected that this highlight on the differences in the metabolome profiles of PVV and PVF could lead to potential research on the pharmacological effect of PVF, as well as the differences in pharmacological activities of these two varieties.

**Figure 6:**
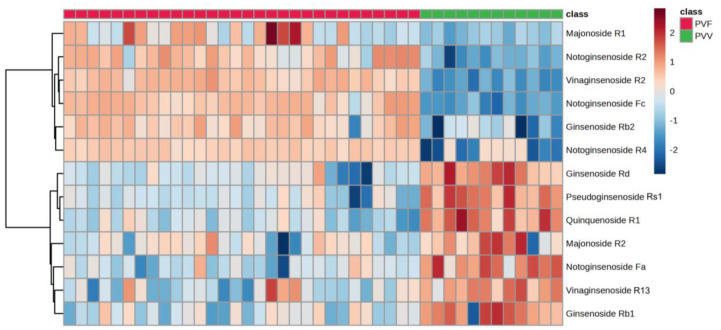
Thirteen differentially expressed ginsenosides formed two distinct clusters according to their expression patterns in the training set.

Nonetheless, the distinct ginsenoside levels suggested that using ginsenosides alone could still be sufficient to classify PVV and PVF (Figure 6).

### 3.4. Characterization of the Potential Differential Markers Using ROC Curve-Based Modeling

**Figure 7:**
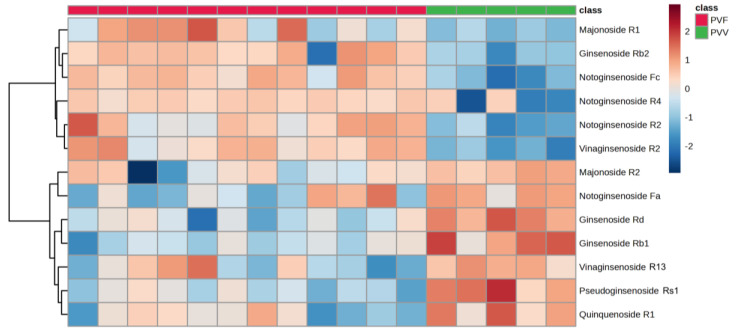
Thirteen differentially expressed ginsenosides formed two distinct clusters according to their expression patterns in the test set.

We also conducted exploratory data analysis on the test set containing 13 potential biomarker candidates found in the training set. In particular, the heatmap (with feature-based clustering) formed two clusters that matched well with the expression pattern found in the training set (Figure 7). The analysis suggested that it could be feasible to differentiate PVV and PVF samples.

**Figure 8:**
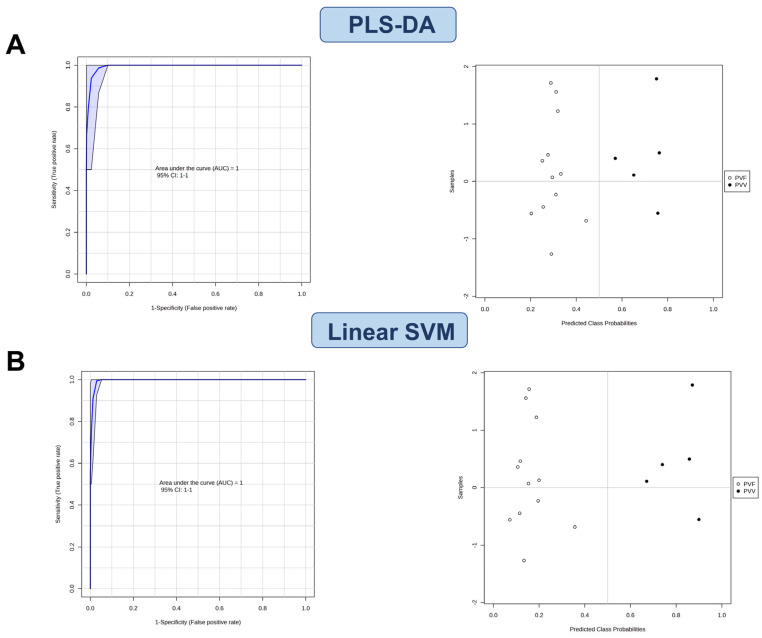
ROC analysis using (**A**) PLS-DA and (**B**) linear SVM models indicated distinct ginsenoside profiles of PVV and PVF.

Indeed, the ROC curve-based modeling using either PLS-DA (two latent variables) or linear SVM showed perfect classification (area under the curve (AUC) = 1.00, 95% confidence interval = 1.00–1.00) (Figure 8A,B). Collectively, our study found that PVV and PVF had distinct metabolic and ginsenoside profiles.

## 4. Conclusions

In this study, an untargeted metabolomic fingerprinting strategy incorporating UPLC-QTOF-MS, multivariate analysis, and machine learning method was developed, with the objective of identifying biomarkers for the authentication of *Panax vietnamensis* var. *vietnamensis* and *Panax vietnamensis* var. *fuscidiscus*. All collected samples were characterized by ITSr-DNA sequence to confirm their correct origins. Next, 13 ginsenosides markers were selected using PLS-DA model based on the UPLC- QTOF-MS analysis, resulting in the clear separation between two groups. The differentiation model also showed great match in expression pattern when being externally validated by test set. This is the first study to systematically compare and highlight the differences between PVV and PVF metabolomes. Considering the morphological similarity of and little available information on the chemical differences between these two varieties, which is essential for authentication practice, the developed approach in our study reveals highly effective discrimination, hence proving its promising application in the quality control of raw material and natural products derived from PVV and PVF.

## Figures and Tables

**Table 1:** The differentially expressed ginsenosides (unpaired *t*-test) between PVV and PVF groups and the corresponding VIP score from the optimal PLS-DA model.

Ginsenoside	Fold Change (PVV/PVF)	*p*-Value	False-Discovery Rate	VIP Score
**Ginsenoside Rb1**	2.00	1.20E-05	3.15E-05	0.71
**Ginsenoside Rb2**	0.12	5.03E-08	2.39E-07	1.62
**Ginsenoside Rd**	2.23	3.77E-08	1.85E-07	0.87
**Majonoside R1**	0.31	3.47E-05	8.18E-05	0.84
**Majonoside R2**	1.39	5.31E-03	0.008724	0.41
**Notoginsenoside Fa**	6.62	5.83E-09	3.47E-08	1.37
**Notoginsenoside Fc**	0.03	9.39E-21	8.35E-19	2.07
**Notoginsenoside R2**	0.08	5.57E-13	1.03E-11	1.65
**Notoginsenoside R4**	0.20	8.89E-09	5.08E-08	2.09
**Pseudoginsenoside Rs1**	8.54	3.43E-10	2.70E-09	1.49
**Quinquenoside R1**	4.99	1.54E-11	1.81E-10	1.30
**Vinaginsenoside R13**	1.56	1.08E-05	2.87E-05	0.62
**Vinaginsenoside R2**	0.13	2.27E-24	1.45E-21	1.58

## Data Availability

The data presented in this study are available in the main article and the supplementary materials.

## Supplementary Materials

The following supporting information can be downloaded at: https://www.mdpi.com/article/10.3390/metabo13060763/s1, Figure S1: *Panax vietnamensis* var. *vietnamensis* roots; Figure S2: *Panax vietnamensis* var. *fuscidiscus* roots; Figure S3: The schematic view of this study’s workflow; Figure S4: The phylogenetic relationship of all specimens with other species in the *Panax* genus using ML method; Table S1. Compounds identified in PVV and PVF.

## Sample Availability

Samples of the compounds *Panax vietnamensis* var. *vietnamensis* and *Panax vietnamensis* var. *fuscidiscus* are available from the authors.
