# Supplementary material for: Untargeted Metabolomics Approach for the Differentiation between Panax vietnamensis var. vietnamensis and Panax vietnamensis var. fuscidiscus
Source: Metabolites. 2023 Jun 19;13(6):763. doi: 10.3390/metabo13060763 (PMC10301110; doi:10.3390/metabo13060763)
Supplement: Supplementary file 1 [file metabolites-13-00763-s001.zip › metabolites-2427252-supplementary.pdf]

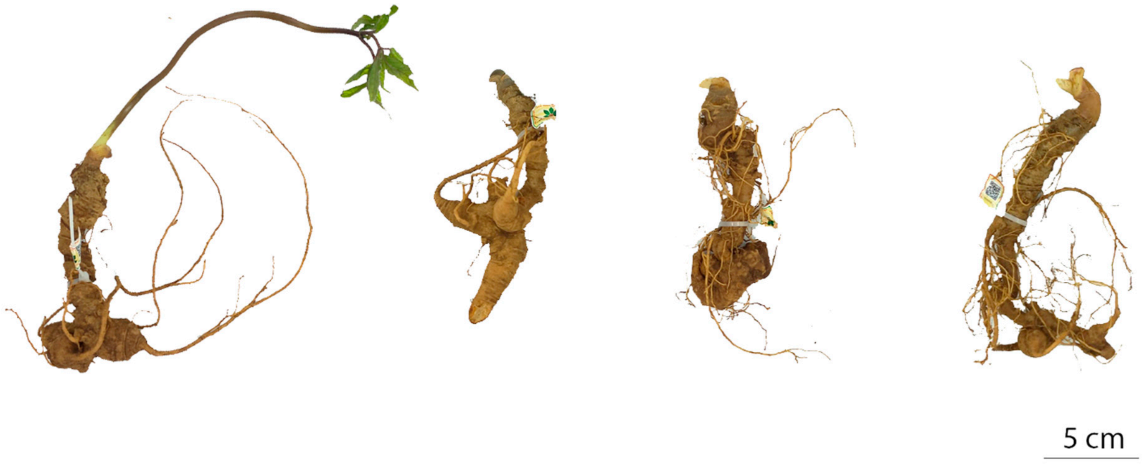

Figure S1. *Panax vietnamensis* roots.

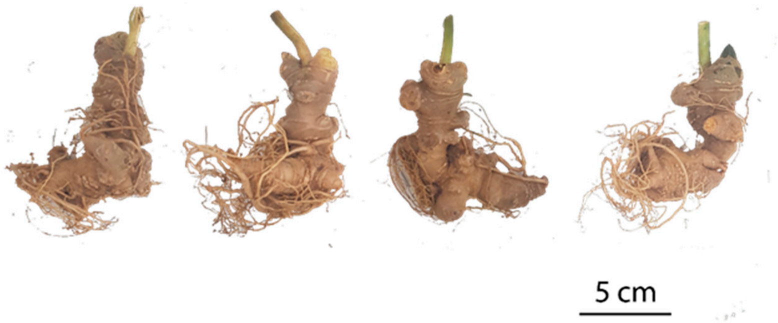

Figure S2. *Panax vietnamensis* var *fuscidiscus* roots.

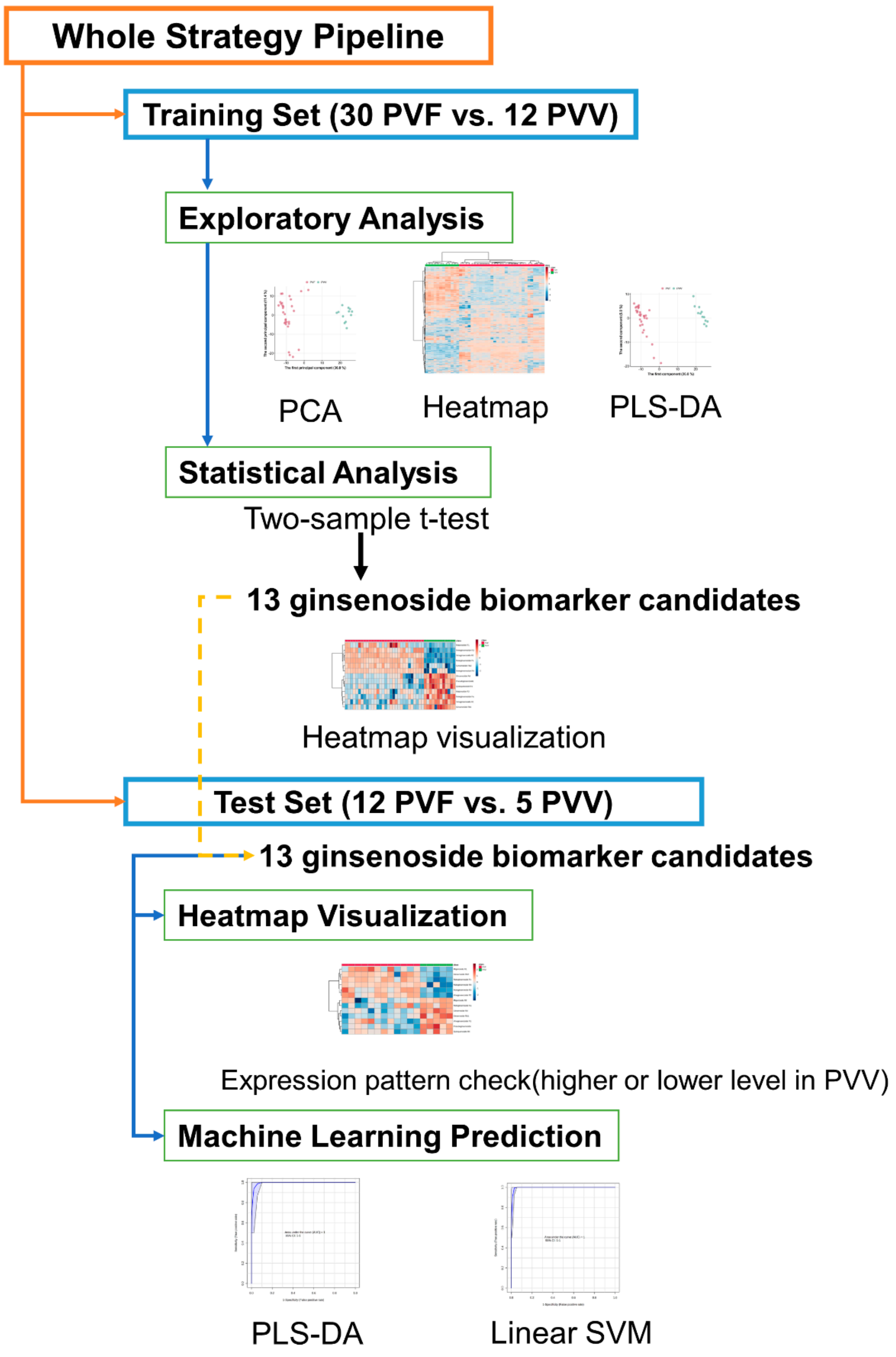

Figure S3. The schematic view of this study's workflow.

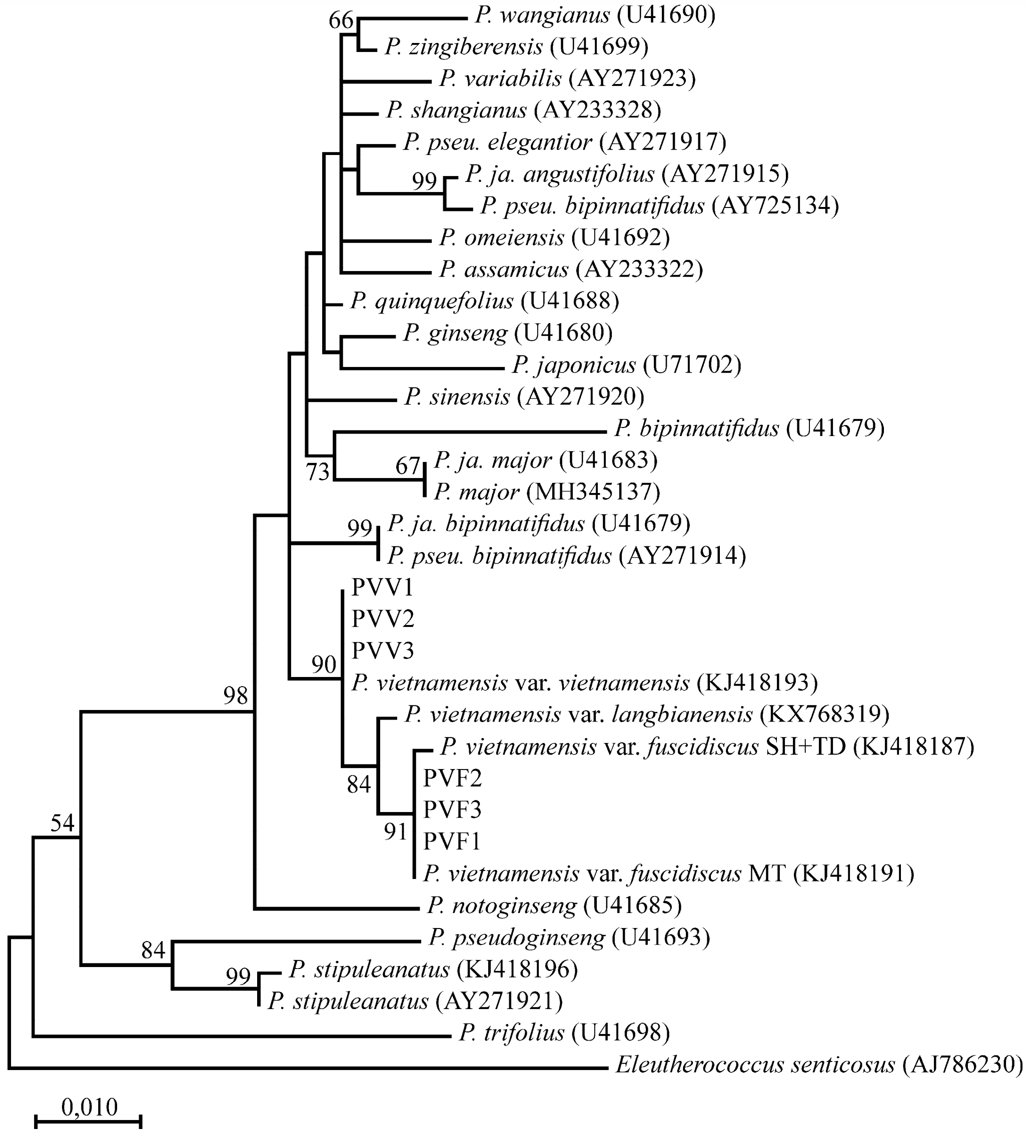

Figure S4. The phylogenetic relationship of the all specimens with other species in the *Panax* genus using ML method. The number in the brackets are Genbank accession number. The number in the root are bootstrap support after 500 replicates.

Table S1. Compounds indentified in PV and PVF

Number	Compounds	Rt (min)	[M-H] (*m*/*z*)	PVV	PVF	Absolute	Tentative
**1**	Vinaginsenoside R13	2.04	71.4366	x	x		x
**2**	Vinaginsenoside R14	4.59	801.4635	x	x		x
**3**	Vinaginsenoside R4	9.11	961.5378	o	x		x
**4**	Majonoside R1	10.28	815.4793	x	x	x	
**5**	Notoginsenoside R1	11.26	931.5252	x	x	x	
**6**	Ginsenoside Rg1	16.38	845.4894	x	x	x	
**7**	Majonoside R2	17.40	785.4688	x	x	x	
**8**	Pseudoginsenoside Rt4	20.15	653.4340	x	x	x	
**9**	Vinaginsenoside R11	21.87	785.4680	x	x	x	
**10**	Hemsloside Ma3	29.42	1087.5281	o	x		x
**11**	Pseudoginsenoside Rc1	31.61	987.5527	x	x		x
**12**	Vinaginsenoside R2	32.71	827.4795	x	x	x	
**13**	Notoginsenoside R2	34.36	769.4752	o	x		x
**14**	Notoginsenoside R4	36.36	1239.6357	o	x		x
**15**	Notoginsenoside Fa	38.13	1239.6364	x	o		x
**16**	Ginsenoside Rb1	40.60	1107.5946	x	x	x	
**17**	Quinquenoside R1	43.11	1149.6049	x	o		x
**18**	Notoginsenoside Fc	44.43	1209.6281	o	x		x
**19**	Quinquenoside R1 isomer	45.81	1149.6046	o	x		x
**20**	Ginsenoside Rb2	46.81	1077.5844	o	x	x	
**21**	Ginsenoside Rd	49.64	945.5427	x	x	x	
**22**	Pseudoginsenoside Rs1	50.05	987.5540	x	x		x
